# 5-Aminolevulinic Acid and Soil Fertility Enhance the Resistance of Rosemary to *Alternaria dauci* and *Rhizoctonia solani* and Modulate Plant Biochemistry

**DOI:** 10.3390/plants8120585

**Published:** 2019-12-09

**Authors:** Hosam O. Elansary, Diaa O. El-Ansary, Fahed A. Al-Mana

**Affiliations:** 1 Plant Production Department, College of Food and Agricultural Sciences, King Saud University, P.O. Box 2455, Riyadh 11451, Saudi Arabia; falmana@ksu.edu.sa; 2 Floriculture, Ornamental Horticulture, and Garden Design Department, Faculty of Agriculture (El-Shatby), Alexandria University, Alexandria 21526, Egypt; 3 Department of Geography, Environmental Management, and Energy Studies, University of Johannesburg, APK campus, Johannesburg 2092, South Africa; 4 Precision Agriculture Laboratory, Department of Pomology, Faculty of Agriculture (El-Shatby), Alexandria University, Alexandria 21526, Egypt; diaaagri@hotmail.com

**Keywords:** 5-aminolevulinic acid, *Alternaria dauci*, *Rhizoctonia solani*, *Salvia rosmarinus*, gene expression

## Abstract

Fungal infection of horticultural and cereal crops by *Alternaria dauci* and *Rhizoctonia solani* represents an important biotic stress that could be alleviated by application of 5-aminolevulinic acid (ALA) to fertile and poor soils. Therefore, in this study, the morphological, physiological, biochemical, and genetic effects of ALA application (eight weekly applications at 3–10 ppm) to *A. dauci-* and *R. solani-*infected *Salvia rosmarinus* (rosemary) in fertile and poor soils were investigated. ALA-treated plants produced the longest and highest number of branches and had higher fresh and dry weights. There were increases in the major essential oil constituents (1,8-cineole, linalool, camphor, and borneol), as shown by Gas chromatography–mass spectrometry (GC-MS); higher antioxidant activities in DPPH and β-carotene-bleaching assays; upregulated superoxide dismutase (SOD) and catalase (CAT) antioxidant enzyme activities; increased total phenolics, chlorophyll, soluble sugars, and proline; increased gas exchange parameters; enhanced leaf water potential and relative water content (RWC); and upregulated expression of *DREB2* and *ERF3* (stress-related genes) and *FeSOD*, *Cu/ZnSOD,* and *MnSOD* (antioxidant genes). Several mechanisms were involved, including stress tolerance, antioxidative, and transcription regulation mechanisms. Furthermore, ALA performance was increased in higher-quality soils with higher nutrient content. This study demonstrated the novel application of ALA as a biotic stress ameliorant with enhanced performance in fertile soils.

## 1. Introduction

5-Aminolevulinic acid (ALA) is a natural compound found in plants that is used internally as a precursor of chlorophylls and phytochromes [1]. ALA has proven effects as an abiotic stress ameliorant against salinity, drought, cold, and heat [2,3,4], with other applications in agriculture, such as fruit coloring [5], fruit thinning [6], and ion uptake [7]. This natural compound, when applied externally to plant leaves, stimulates the photosynthetic apparatus and gas exchange, leading to enhanced stress tolerance during abiotic stress. Some mechanisms have been indicated in these effects, including the osmotic adjustment and antioxidative mechanisms. However, a complete picture of the different mechanisms involved and an in-depth understanding of the interactions of the known mechanisms are still lacking [8]. Furthermore, the effects of ALA as a stress ameliorant during biotic stress conditions have not yet been explored.

*Alternaria dauci* (Pleosporaceae) is a common plant pathogen that infects most horticultural crop leaves and causes water-soaked leaf spot, yellowing of leaves, and leaf death. It can also cause seedling damping-off, seed stalk blight, and infection of the inflorescence. Dozens of crops are regularly infected worldwide, including lettuce, carrot, and rosemary, resulting in significant economic losses [9]. *Rhizoctonia solani* (Ceratobasidiaceae) is a common plant pathogen that attacks the roots and stems of seeds, young plants (seedlings), and mature plants and causes several diseases, including damping-off, root rot, and wire stem. *R. solani* causes severe losses in all herbaceous plants, including major economic cereals in Europe, North and South America, and Australia and in vegetable crops, including potatoes, tomatoes, and cabbage. The control of such diseases is sophisticated, and an ideal control is not possible [10,11]; however, regular control is dependent on a mixture of factors, including the host crop, the environment, and the pathogen itself. Developing novel tools and strategies for fungal disease control in horticultural crops, such as using ALA, is critical for the development of agricultural production and industries.

*Salvia rosmarinus* (Lamiaceae) is an aromatic perennial herb, commonly known as rosemary. It is native to the Mediterranean region and has been grown successfully since ancient times [12]. The plant stems and leaves are harvested and consumed fresh or dry. The stems, leaves, and flowers are used for the extraction of the essential oil, which is routinely used in the perfume, pharmaceutical, and food industries. Fresh and dried plants are traditionally used for the treatment of several diseases, and recent studies have also revealed a large array of activities, including antioxidant, antimicrobial, and anticancer effects [13,14]. The plant is susceptible to various fungal infections, including *A. dauci* and *R. solani,* and no studies have been performed on the alleviation of biotic stress in rosemary using 5-aminolevulinic acid.

The current study aimed to explore the possible use of ALA as a biotic stress ameliorant against *A. dauci* and *R. solani* in rosemary. The morphological, physiological, biochemical, and genetic performances of the treated plants were investigated. Additionally, a novel approach was explored by using ALA as a biotic stress ameliorant under high and low fertility conditions. Several mechanisms were identified in these activities and are discussed here.

## 2. Results

### 2.1. Vegetative Growth

**Figure 1:**
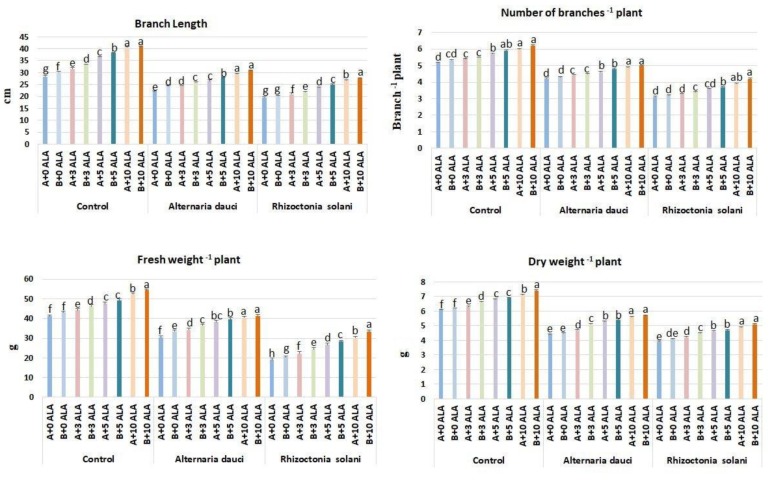
Rosemary mean morphological parameters following 5-aminolevulinic acid (ALA) treatment (3, 5, and 10 ppm) and *Alternaria dauci* and *Rhizoctonia solani* inoculations in different soils (A and B). Different letters within treatment indicate significant differences.

The effects of ALA, soil type, and fungal infection on the rosemary plant’s vegetative growth are shown in Figure 1. Two different media were used: A (30% sand + 70% loam) and B (100% loam soil). The biotic stress produced shorter plants with a lower number of leaves and caused reductions in the fresh and dry weights. Plants infected with *A. dauci*/*R. solani* and treated with high doses of ALA (5 and 10 ppm) produced the longest and highest number of branches under different soil mixes. The fresh and dry weights were the highest in infected and healthy plants treated with high doses of ALA (5 and 10 ppm). Healthy plants showed the highest vegetative growth among plants treated with ALA. Soil mix B plants showed higher performance than mix A under all growing conditions.

### 2.2. Essential Oil Composition

Four major essential oil constituents were identified: 1,8-cineole, linalool, camphor, and borneol, as shown in Table 1. The biotic stress produced higher amounts of linalool, camphor, and borneol compared to the control. In the control treatments, the application of ALA increased the content of these four major constituents, and soil mix B showed no significant difference from mix A. Under fungal infection, the application of higher doses of ALA yielded significant enhancement in the content of the major essential oil constituents.

### 2.3. Antioxidant Activities

**Figure 2:**
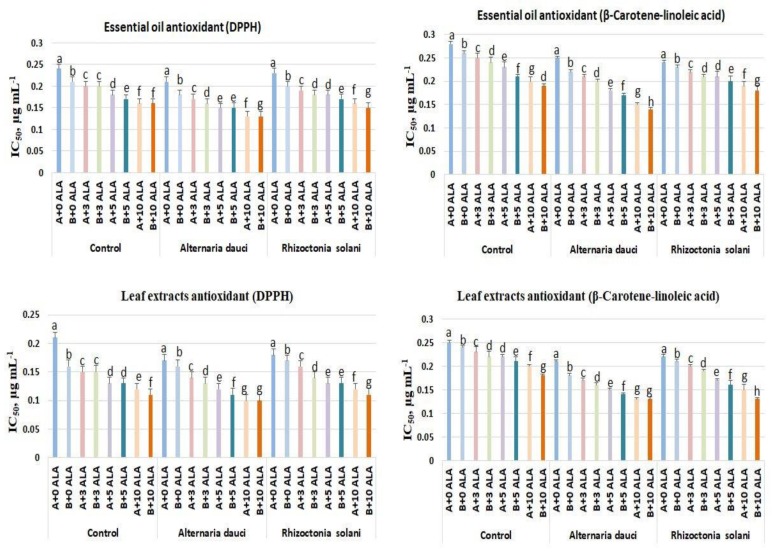
Total antioxidant activities of rosemary essential oils and leaf extracts following ALA treatment (3, 5, and 10 ppm) and *Alternaria dauci* and *Rhizoctonia solani* inoculations in different soils (A and B). Different letters within treatment indicate significant differences.

**Figure 3:**
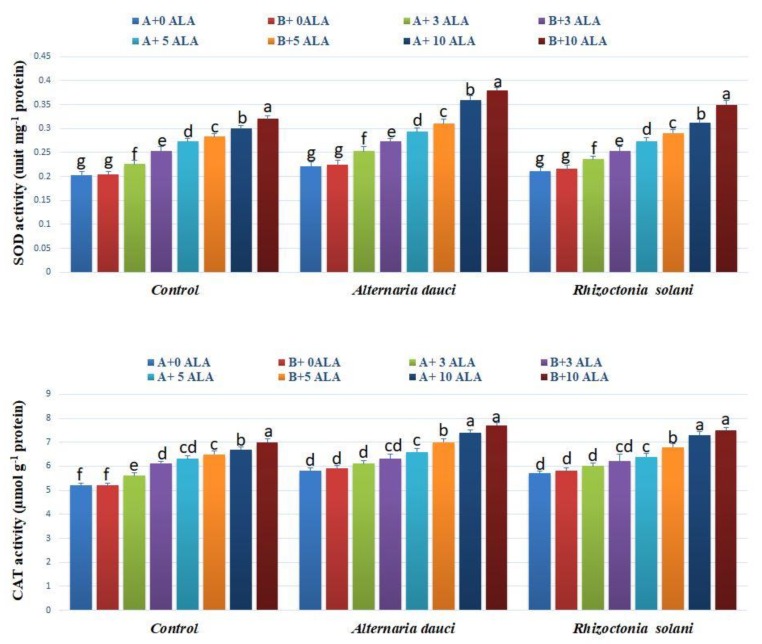
Catalase (CAT) and superoxide dismutase (SOD) enzymes activities in rosemary plants subjected to ALA treatment (3, 5, and 10 ppm) and *Alternaria dauci* and *Rhizoctonia solani* inoculations in different soils (A and B). Data are means ± SD (*n* = 3). Different letters within treatment indicate significant differences.

The essential oil and leaf extract antioxidant activities are shown in Figure 2. Under control conditions, ALA treatment and soil mix B showed the highest antioxidant activities (lower values of IC_50_) in both essential oils and leaf extracts in the DPPH and β-carotene-linoleic acid assay. The fungus-infected plant essential oils and leaf extracts showed lower total antioxidant activities (higher values of IC_50_) compared to infected plants treated with ALA. Superoxide dismutase (SOD) and catalase (CAT) enzyme activities were higher in the leaves of plants treated with ALA for both healthy and infected plants, as shown in Figure 3. Soil mix B showed higher SOD activity in leaf extracts as compared to soil mix A.

### 2.4. Chlorophyll, Soluble Sugars, and Proline Compositions

**Figure 4:**
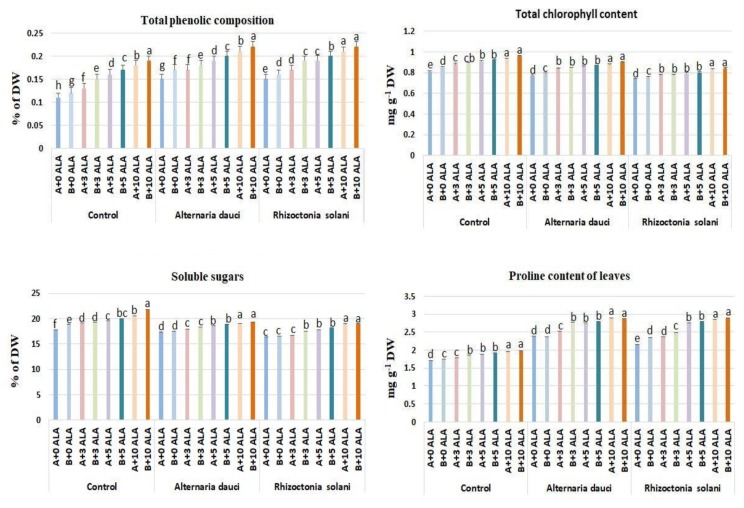
The mean of total phenolic, chlorophylls, carbohydrates, and proline in rosemary leaves following ALA treatment (3, 5, and 10 ppm) and *Alternaria dauci* and *Rhizoctonia solani* inoculations in different soils (A and B). Different letters within treatment indicate significant differences.

Total phenolics, chlorophyll, carbohydrates, and proline were determined in the leaves, as shown in Figure 4. Under control conditions, ALA caused increases in phenolics as compared to untreated plants. Plants infected with *A. dauci*/*R. solani* and treated with ALA showed higher values of total phenolics compared to ALA-untreated plants. Total chlorophyll was higher in ALA-treated plants in the control as well as in the infected plants. Carbohydrates were higher in infected plants treated with ALA as compared to untreated plants. Proline accumulated in larger amounts in the leaves of infected plants treated with ALA as compared to the control.

### 2.5. Gas Exchange and Water Measures

**Figure 5:**
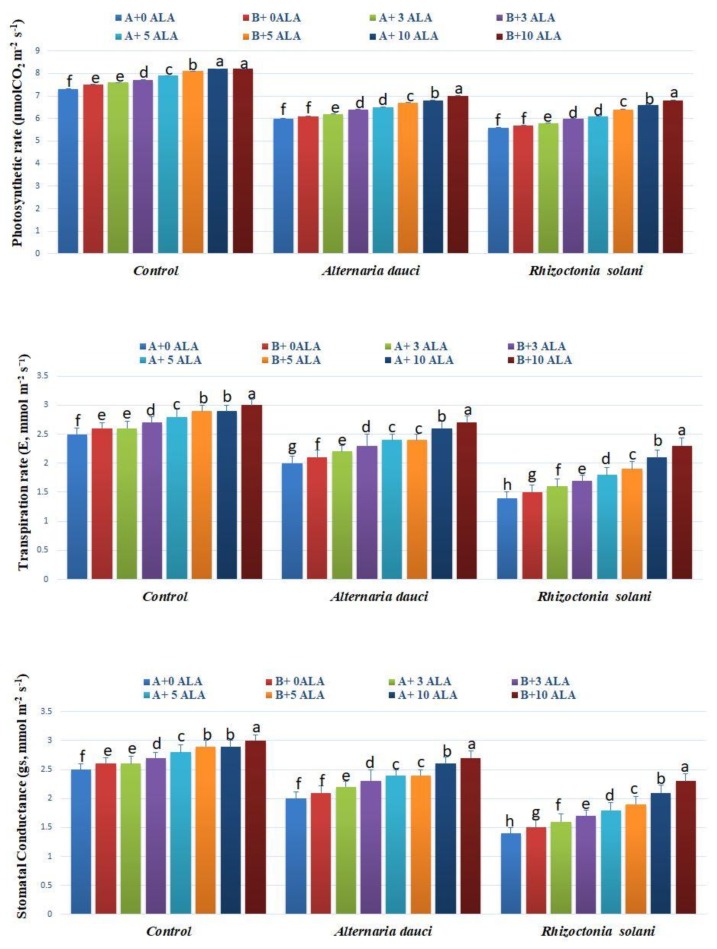
Mean values of rosemary photosynthetic rate (*P_n_*, μmol CO_2_ m^−2^ s^−1^), transpiration rate (*E*, mmol m^−2^ s^−1^), and stomatal conductance (*g_s_*, mmol m^−2^ s^−1^), as affected by ALA treatment (3, 5, and 10 ppm) and *Alternaria dauci* and *Rhizoctonia solani* inoculations in different soils (A and B). Different letters within treatment indicate significant differences.

**Figure 6:**
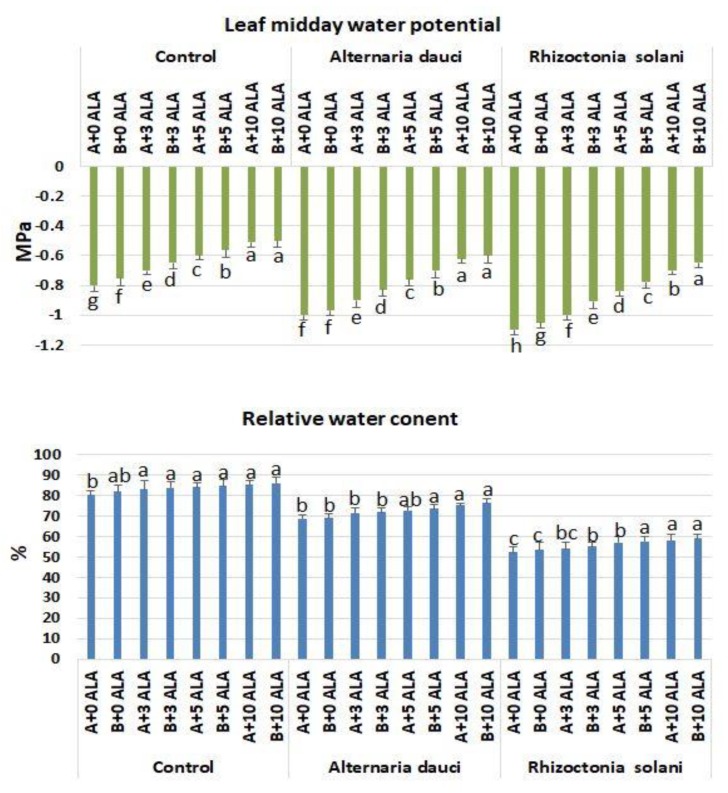
Leaf midday water potential and relative water content in rosemary plants subjected to ALA treatment (3, 5, and 10 ppm) and *Alternaria dauci* and *Rhizoctonia solani* inoculations in different soils (A and B). Data are means ± SD (*n* = 3). Different letters within treatment indicate significant differences.

The gas exchange parameters of the experiment are shown in Figure 5. The photosynthetic and transpiration rates and the conductance were higher in plants subjected to higher doses of ALA under normal growing conditions. Fungus-infected plants showed higher levels of photosynthesis and transpiration as well as stomatal conductance when subjected to ALA, as compared to untreated plants. Leaf water potential and relative water content (RWC) were higher also in ALA-treated plants under control conditions and in plants subjected to fungal infections (Figure 6). Soil mix B showed higher patterns of gas exchange as compared to soil mix A.

### 2.6. Gene Expression

**Figure 7:**
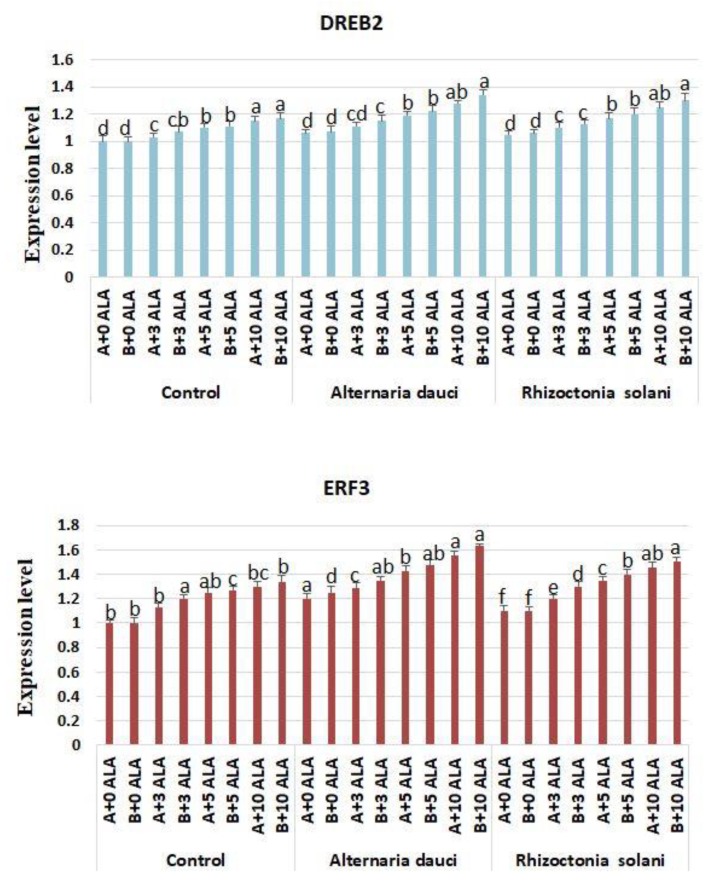
Gene expression levels of *DREB2* and *ERF3*(C) in rosemary plants subjected to ALA treatment (3, 5, and 10 ppm) and *Alternaria dauci* and *Rhizoctonia solani* inoculations in different soils (A and B). Data are means ± SD (*n* = 3). Different letters within treatment indicate significant differences.

**Figure 8:**
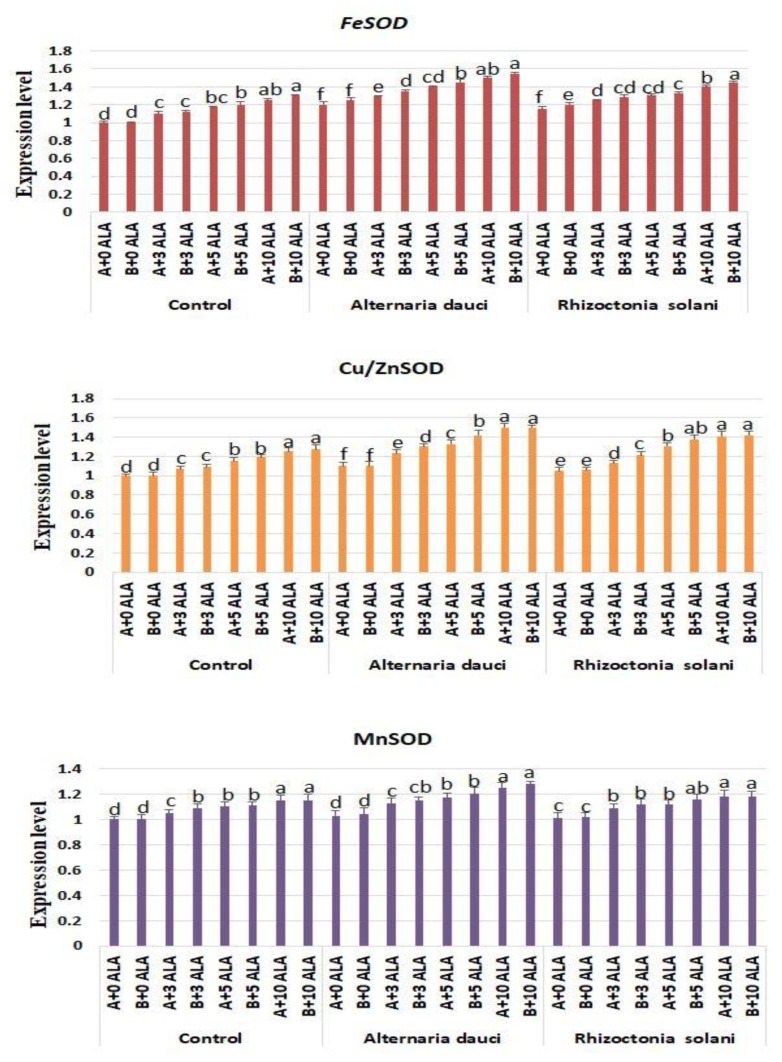
Gene expression levels of *FeSOD, Cu/ZnSOD*, and *MnSOD* in rosemary plants subjected to ALA treatment (3, 5, and 10 ppm) and *Alternaria dauci* and *Rhizoctonia solani* inoculations in different soils (A and B). Data are means ± SD (*n* = 3). Different letters within treatment indicate significant differences.

The gene expression of control plants, as well as infected plants, during ALA treatment is shown in Figure 7 and Figure 8. Both *DREB2* and *ERF3* stress-related gene expression patterns showed significant increases under ALA treatment in both control and infected plants. The highest values were achieved when applying higher doses of ALA. Expression of antioxidant genes *FeSOD*, *Cu/ZnSOD*, and *MnSOD* was higher in ALA-treated plants under all conditions, and this expression was elevated in infected plants.

## 3. Discussion

Several reports have indicated that ALA might increase the fresh and dry weight of plants during stress conditions [2,4]. However, other reports indicate that ALA might act as a vegetative growth retardant with herbicidal activity and cause leaf necrosis [15,16]. The present study found stimulatory morphological effects of ALA in rosemary plants, including increased length, number of branches, and fresh and dry weights, which have not been previously reported in this species. The improved vegetative growth of plants infected with fungi as a biotic stressor and the higher ALA activity with increasing soil fertility or improvement in physiochemical properties (as in soil mix B) are novel findings. These could be attributed to the increased photosynthetic rates in plants treated with high doses of ALA. ALA stimulates vegetative growth by stimulating photosynthesis and photosynthetic rates [3]. Fungal infection by *A. dauci* and *R. solani* reduced the growth of infected plants that were not treated with ALA (Figure 1). The application of ALA alleviated this biotic stress and enhanced vegetative growth; to our knowledge, the present study is the first to report this finding. Furthermore, the availability of macro- and micronutrients in the soil increased the performance of ALA, as expressed in increased fresh and dry weights [8].

Rosemary plants are characterized by the presence of 1,8-cineole, linalool, camphor, and borneol in the essential oil of the leaves [12], as found in this study. Treatment with high doses of ALA significantly increased the ratio of the main components in the essential oil of the leaves in plants showing increased vegetative growth due to alleviated biotic stress. The change in major components of the essential oil could be attributed to the regulatory effects of ALA on plant metabolism, identified here as increased gas exchange and leaf water potential and relative water content, soluble sugars, and phenols. Previous investigations have found that ALA can modulate leaf water potential and gas exchange under abiotic stress conditions [4]. The shifts in essential oil composition of the leaves help to control fungal infections and increase the resistance of infected plants. Most of these main constituents have shown antifungal activities against *A. dauci* and *R. solani* [17,18,19].

ALA is known to influence the antioxidant mechanism of treated plants during specific stress conditions, such as drought, salinity, and cold, by increasing the photosynthetic rate, reducing damage to the photosynthetic apparatus, enhancing gas exchange, and increasing chlorophyll content [20,21,22]. The antioxidant activities of the essential oils and leaf extracts were higher in ALA-treated plants than in the control because of the increased SOD and CAT enzyme activities in the leaves, as well as the increase in primary essential oil constituents exhibiting strong antioxidant activities, such as cineol and linalool [23,24]. Such increases in the antioxidant defense mechanism associated with increased growth of plants and alleviation of fungal infection stress have not been previously reported. In addition, there was an accumulation of proline, which is known to accumulate under stress conditions in most plants. Proline is the antioxidant defense mechanism molecule that maintains turgor pressure and membrane stability.

Increases in the total phenolic composition of the leaves were found, and this accumulation played a major role in increasing the antioxidant activity of the leaves and resistance to biotic and abiotic stresses. Previous reports have indicated that ALA application can increase the phenolic composition of treated plants [4,25]. The present study showed that soil fertility might increase phenolic and antioxidant accumulation in the leaves of ALA-treated plants under biotic stress. Previous studies found that ALA application enhances nutrient uptake under normal and abiotic stress conditions [7,26]. The present study confirmed that this occurs under biotic stress conditions as well. There were increases in the chlorophyll composition of leaves in plants treated with ALA, which is the precursor of chlorophyll and other important molecules in plants [1]. The accumulation of soluble sugars found here under biotic stress might be considered a stress tolerance mechanism because this accumulation enhanced the osmotic adjustment and performance of the plants subjected to fungal infection. The accumulation of sugars was higher in loamy soil subjected to ALA, owing to the higher rate of photosynthesis in the leaves and levels of macro- and micronutrients in the soil. The increases in gas exchange parameters in ALA-treated plants were associated with increased leaf water potential and RWC under fungal infection, which play a major role in the improvement of vegetative growth of the plants and are considered a stress tolerance mechanism [27]. The treatment with ALA can recover the transpiration rate and the stomatal conductance when the plants are attacked by *Alternaria*, with respect to the non-attacked plants.

The present study also showed increases in the gene expression of stress-related (*DREB2* and *ERF3*) and antioxidant genes (*FeSOD*, *Cu/ZnSOD*, and *MnSOD*) in plants treated with ALA under biotic stress conditions as compared to the control. Previous studies have indicated that ALA upregulates the expression of POD, CAT, and ascorbate peroxidase (APX) in *Brassica napus* subjected to salt stress conditions [21]. However, the stimulatory activity of these genes had not previously been reported under biotic stress conditions, as in this study. Furthermore, these activities explain the enhanced antioxidant properties of ALA-treated plants and indicate that the antioxidant mechanism might be considered the main function of ALA as a biotic stress ameliorant. It was clear that soil mix B (100% loam) yielded better morphological and physiological performance than soil mix A (70% loam + 30% sand).

In the current study, we demonstrated the novel finding that ALA application alleviates plant biotic stress conditions via several mechanisms, including stress tolerance and the antioxidative mechanism, by enhancing relative water content gas exchange and osmotic adjustment, activating antioxidant enzymes, accumulating antioxidants, and stimulating stress and antioxidant genes. Furthermore, ALA performance was increased in higher-quality soils with higher nutrient levels.

## 4. Material and Methods

### 4.1. Plant Material, Soil Mixes, ALA Treatment, and Inoculum Preparation

Young *Salvia rosmarinus* (L.) Schleid. plantlets (10 cm) containing 4–5 buds were obtained from local commercial nurseries in two successive seasons (January 2018 and 2019). The plants were identified, and voucher specimens were maintained at Alexandria University, Egypt. Plants were transplanted into larger 3.1 L plastic pots containing two different media: A (30% sand + 70% loam) and B (100% loam soil). The physiochemical properties of both soils are shown in Table 2. Organic carbon was determined, following Reference [28]. Fe, Cu, Zn, and Mn were determined by atomic absorption spectrophotometry (AASVario 6, Jena, Germany) [29]. Nitrogen was determined using the Kjeldahl method [30] and phosphorus was determined via the phosphomolybdic acid method [31]. Experiments were conducted in a greenhouse and repeated during the years 2018 and 2019. Growing media were supported by 2g L^−1^ fertilizer (Crystalon^®^, 19% N: 19% P: 19% K). The mean growing temperatures were 15.1 °C at night a 24.5 °C during the day, relative humidity ranged between 68% and 82%, and photosynthetic active radiation was 900 W m^−2^ at 12:00. The plants were watered every 48 h until drop-off. A foliar spray of 5-aminolevulinic acid (ALA, Sigma-Aldrich, Berlin, Germany) at 3, 5, and 10 ppm was applied weekly until drop-off. Untreated plants were considered the control.

*A. dauci* and *R. solani* fungi were isolated from infected rosemary plants showing symptoms of blight and crown rot. The plant surface was sterilized with 0.1% sodium hypochlorite, and small infected pieces of leaves or roots were cut and placed in Petri dishes with potato dextrose agar medium for 2 weeks at 25 °C. The fungi were identified using microscopic morphological characteristics. The inoculum of each fungus was prepared by introducing a sterile needle into sterilized and filtered flasks of Richard’s liquid medium, containing 5 g KH_2_PO_4_, 10 g KNO_3_, 2.5 g MgSO_4_, 0.02 g FeCl_3_, 50 g sucrose, and 1000 mL distilled water. They were then incubated for 2 weeks at 25 ± 1 °C, and the mycelia mat was isolated by filtering with filter paper No. 1, washing with distilled water, mixing 10 g of mycelium in 100 mL distilled water, blending the mix at 10,000 rpm in a Waring blender, and inoculating a suspension of 1 g fungus suspension to the young plants. Untreated plants with fungi were used as a control. Factorial experimental design was used. Three blocks were used: Each block contained 24 treatments (3 fungal treatments, including control × 2 soil mixes × 4 ALA doses). Each treatment was represented by 6 replicates, and the total number of plants was 144 plants per block. All chemicals were HPLC grade (Sigma-Aldrich, Cairo, Egypt).

### 4.2. Morphological Measurements

After 8 weeks, branch length (cm) was determined, number of branches per plant was counted, and the fresh (FWs, g) and dry weights (DWs, g) were obtained. The dry weight was determined by preliminary drying at room temperature (20 °C), then dried in the oven at 30 °C until reaching a constant weight, following Reference [32].

### 4.3. GC-MS of Essential Oil and Preparation of Leaf Extracts

Leaf essential oil was extracted via hydrodistillation of dried leaves (500 g) using a Clevenger-type apparatus. The oil was dried, filtered, and kept at 4 °C under dark conditions [33]. A gas chromatograph mass spectrometer (Thermo Scientific, Waltham, MA, USA) equipped with a TG-1MS column was used for the analyses. The temperature was ramped from 45 °C to 165 °C at 4 °C min^−1^ for 2 min, followed by a gradual increase reaching 280 °C for 15 min. Helium was used as a carrier gas, and the same temperature was used for gas chromatography with flame-ionization detection (GC-FID). The retention times and indices of *n*-alkanes (C_10_–C_36_) were used for the identification of detected compounds along with computer matching in the National Institute of Standards and Technology (NIST) mass spectra ver. 2.0 and WILEY libraries. Literature references were also used for accurate identification and comparison [12,34]. Leaf extracts were obtained as described previously in [35] and then maintained at −80 °C for further analysis.

### 4.4. Antioxidant Activities

The total antioxidant activities were determined for leaves using the 2,2-diphenyl-1-picrylhydrazyl (DPPH) and β-carotene-linoleic acid assays [33]. Briefly, for DPPH, 50 μL (1 mg·mL^−1^) of the leaf methanolic extract was mixed with DPPH solution (5 mL, 0.004%) and incubated for 30 min in the dark, and then the absorbance was determined at 517 nm in a spectrophotometer. For the β-carotene-linoleic assay, β-carotene was incubated for 48 h with leaf methanolic extract (50 μL), and the absorbance was then determined at 470 nm. The results were expressed as IC_50_ in µg mL^−1^. Superoxide dismutase (SOD) and catalase (CAT) activities were quantified in leaf tissues [12,36]. Experiments were repeated twice in triplicate.

### 4.5. Chlorophyll, Soluble Sugars, and Proline Compositions

Total chlorophyll was determined in leaves, following Reference [37]. The soluble sugar in the leaves was determined (% of DW), following Reference [38], and proline was determined in the leaves, following Reference [39].

### 4.6. Gas Exchange and Water Measures

Net photosynthetic rate (*A*), transpiration rate (*E*), and stomatal conductance (*g_s_*) were determined using portable gas exchange equipment (ADC LCi, Bioscientific Ltd., Hoddesdon, UK) in the morning (10:00) in mature leaves at the end of the experiment. The leaf water potential and relative water content (RWC) of mature leaves were determined at noon (12:00), following References [4,40].

### 4.7. Gene Expression

To assess the expression of stress-related genes, quantitative real-time PCR (qRT-PCR) was conducted for *FeSOD, Cu/ZnSOD, MnSOD, DREB2,* and *ERF3* in three plants of each treatment subjected to ALA and fungal infection. The RNA was isolated from young leaves using the RNeasy Plant Mini Kit (Qiagen, Berlin, Germany). To obtain the cDNA, a Reverse Transcription Kit (Qiagen, Berlin, Germany) was used, and qRT-PCR was performed in triplicate using QuantiTect SYBR Green PCR (Qiagen, Berlin, Germany). PCR primers and conditions followed Reference [12]. The *Actin* housekeeping gene [41] was used as a control, and the 2^−∆∆Ct^ method was used to determine the relative expression levels.

### 4.8. Statistical Analyses

Factorial experimental design was used. Three blocks were used: Each block contained 24 treatments (3 fungal treatments, including control × 2 soil mixes × 4 ALA doses). Each treatment was represented by 6 replicates, and the total number of plants was 144 plants per block. The least significant differences (LSD) were determined for all means (SPSS, PASW Ver. 22, Chicago, IL, USA).

## 5. Conclusions

A novel finding discovered in this study confirmed that ALA could alleviate the biotic stress of rosemary plants caused by *A. dauci*/*R. solani* fungal infections. The biotic stress produced shorter plants with a lower number of leaves and caused reductions in the fresh and dry weights. ALA-treated plants produced the longest and highest number of branches, higher fresh and dry weights, increases in major essential oil components (1,8-cineole, linalool, camphor, and borneol), higher antioxidant activities in DPPH and β-carotene-bleaching assays, upregulated SOD and CAT antioxidant enzyme activities, increased total phenolics, chlorophyll, soluble sugars, and proline, increased gas exchange parameters, leaf water potential, and RWC, and upregulated expression of *DREB2* and *ERF3* (stress-related genes) and *FeSOD*, *Cu/ZnSOD*, and *MnSOD* (antioxidant genes). Several mechanisms were involved, including stress tolerance, antioxidative, and transcription regulation mechanisms. Furthermore, we found that ALA performance was increased in higher-quality soils with higher nutrient content.

## Figures and Tables

**Table 1:** Rosemary mean essential oil composition (2019) following ALA treatment (3, 5, and 10 ppm) and *Alternaria dauci* and *Rhizoctonia solani* inoculations in different soils (A and B). * Different letters within treatment indicate significant differences.

	Soil Type	ALA (ppm)	1,8-cineole RI = 1040	Linalool RI = 1117	Camphor RI = 1139	Borneol RI = 1188
**(control)**	A	0	13.51c *	5.32d	11.02d	10.68d
	B	0	14.12bc	5.49d	11.52cd	10.98cd
	A	3	14.0bc	5.94c	11.53cd	11.21c
	B	3	14.25b	6.23b	11.89c	11.43c
	A	5	15.34ab	6.12b	12.23b	12.11b
	B	5	15.71a	6.35b	12.61b	12.59ab
	A	10	15.82a	7.12a	13.19a	12.86a
	B	10	15.91a	7.35a	13.76a	12.98a
***Alternaria dauci***	A	0	14.53c	6.24d	13.14d	12.14b
	B	0	14.92c	6.35d	13.35cd	12.26b
	A	3	15.04bc	7.46c	13.59c	13.34ab
	B	3	15.61b	7.51c	13.75c	13.45a
	A	5	16.11ab	8.24b	14.22b	13.61a
	B	5	16.61a	8.41ab	14.54a	13.75a
	A	10	16.91a	8.56a	14.86a	13.88a
	B	10	16.85a	8.74a	14.94a	13.91a
***Rhizoctonia solani***	A	0	13.23d	5.84e	12.02c	11.01c
	B	0	13.62c	6.03e	12.66c	11.32c
	A	3	14.08c	6.54d	13.43b	12.32b
	B	3	14.52bc	6.68d	13.79b	12.45b
	A	5	15.04b	7.11c	14.31a	12.85b
	B	5	15.64a	7.46b	14.46a	13.34a
	A	10	15.81a	7.71a	14.64a	13.81a
	B	10	16.14a	8.01a	14.84a	13.85a

**Table 2:** Physiochemical properties of soils used. A (30% sand + 70% loam) and B (100% loam soil).

Composition	Loam + 30% Sand	Loam	LSD *p = 0.05*
pH EC (mmho cm^−1^) Water holding capacity (%) Soil organic carbon (SOC, %)	8.1 0.954 33.5 0.41	8 0.986 45.1 0.52	0.12 0.31 2.2 0.02
Nitrogen (N, g ha^−1^)	162 × 10^3^	190 × 10^3^	3.5
Phosphorus (P_,_ g ha^−1^)	15.69 × 10^3^	18.4 × 10^3^	1.2
Potassium (K_,_ g ha^−1^)	146.2 × 10^3^	199.5 × 10^3^	3.2
Iron (Fe, mg kg^−1^)	4.1	4.6	0.1
Copper (Cu, mg kg^−1^)	0.35	0.42	0.05
Zink (Zn, mg kg^−1^)	3.5	5.1	0.06
Manganese (Mn, mg kg^−1^)	2.8	2.9	0.05
