# Erratum: Elansary et al., 5-Aminolevulinic Acid and Soil Fertility Enhance the Resistance of Rosemary to *Alternaria dauci* and *Rhizoctonia solani* and Modulate Plant Biochemistry. Plants 2019, *8*, 585

**DOI:** 10.3390/plants9010050

**Published:** 2019-12-31

**Authors:** Hosam O. Elansary, Diaa O. El-Ansary, Fahed A. Al-Mana

**Affiliations:** 1 Plant Production Department, College of Food and Agricultural Sciences, King Saud University, P.O. Box 2455, Riyadh 11451, Saudi Arabia; falmana@ksu.edu.sa; 2 Floriculture, Ornamental Horticulture, and Garden Design Department, Faculty of Agriculture (El-Shatby), Alexandria University, Alexandria 21526, Egypt; 3 Department of Geography, Environmental Management, and Energy Studies, University of Johannesburg, APK campus, Johannesburg 2006, South Africa; 4 Precision Agriculture Laboratory, Department of Pomology, Faculty of Agriculture (El-Shatby), Alexandria University, Alexandria 21526, Egypt; diaaagri@hotmail.com

The authors wish to make the following corrections to their paper [1]. The number of the funding agency in the Funding and Acknowledgments sections is incorrect. The details of the corrections are described below.

1. Funding: The study was funded by King Saud University through the Researchers Supporting Project number (RSP-2019/118).
2. Acknowledgments: The authors thank King Saud University through the Researchers Supporting Project number (RSP-2019/118).

These changes do not affect the conclusion of the paper. The authors would like to apologize for any inconvenience this might have caused.
